# NR2F1 stratifies dormant disseminated tumor cells in breast cancer patients

**DOI:** 10.1186/s13058-018-1049-0

**Published:** 2018-10-16

**Authors:** Elin Borgen, Maria C. Rypdal, Maria Soledad Sosa, Anne Renolen, Ellen Schlichting, Per E. Lønning, Marit Synnestvedt, Julio A. Aguirre-Ghiso, Bjørn Naume

**Affiliations:** 1 Department of Pathology, Oslo University Hospital, Oslo, Norway; 2 Department of Pharmacological Sciences, Icahn School of Medicine at Mount Sinai, New York, NY 10029 USA; 3 Division of Hematology and Oncology, Department of Medicine, Department of Otolaryngology, Tisch Cancer Institute, Black Family Stem Cell Institute, Icahn School of Medicine at Mount Sinai, New York, NY 10029 USA; 4 Department of Surgery, Oslo University Hospital, Oslo, Norway; 5 Department of Oncology, Haukeland University Hospital, Bergen, Norway; 6 Department of Clinical Science, Faculty of Medicine, University of Bergen, Bergen, Norway; 7 Department of Oncology, Oslo University Hospital, Oslo, Norway; 8 Institute of Clinical Medicine, University of Oslo, Oslo, Norway

**Keywords:** Disseminated tumor cells, DTC, Dormancy, NR2F1, Bone marrow, Breast cancer, Occult disease, Micrometastasis

## Abstract

**Background:**

The presence of disseminated tumor cells (DTCs) in bone marrow (BM) is an independent prognostic factor in early breast cancer but does not uniformly predict outcome. Tumor cells can persist in a quiescent state over time, but clinical studies of markers predicting the awakening potential of DTCs are lacking. Recently, experiments have shown that NR2F1 (COUP-TF1) plays a key role in dormancy signaling.

**Methods:**

We analyzed the NR2F1 expression in DTCs by double immunofluorescence (DIF) staining of extra cytospins prepared from 114 BM samples from 86 selected DTC-positive breast cancer patients. Samples collected at two or more time points were available for 24 patients. Fifteen samples were also analyzed for the proliferation marker Ki67.

**Results:**

Of the patients with detectable DTCs by DIF, 27% had ≥ 50% NR2F1^high^ DTCs, chosen a priori as the cut-off for “dormant profile” classification. All patients with systemic relapse within 12 months after BM aspiration carried ≤ 1% NR2F1^high^ DTCs, including patients who transitioned from having NR2F1^high^-expressing DTCs in previous BM samples. Of the patients with serial samples, half of those with no relapse at follow-up had ≥ 50% NR2F1^high^ DTCs in the last BM aspiration analyzed. Among the 18 relapse-free patients at the time of the last DTC-positive BM aspiration with no subsequent BM analysis performed, distant disease-free intervals were favorable for patients carrying ≥ 50% NR2F1^high^ DTCs compared with those with predominantly NR2F1^low^ DTCs (*p* = 0.007, log-rank). No survival difference was observed by classification according to Ki67-expressing DTCs (*p* = 0.520).

**Conclusions:**

Our study translates findings from basic biological analysis of DTC dormancy to the clinical situation and supports further clinical studies of NR2F1 as a marker of dormancy.

**Electronic supplementary material:**

The online version of this article (10.1186/s13058-018-1049-0) contains supplementary material, which is available to authorized users.

## Background

Breast cancer patients may experience relapse and subsequent death from the disease many years after primary treatment. This indicates an ability of occult cancer cells to survive in a non- or slow-proliferating state, retaining a potential for progression and proliferation at a later time point [1, 2]. The window of time represented by such minimal residual disease (MRD) represents a possibility for therapeutic intervention to prevent development of future metastasis rather than treat overt metastasis. However, the biology of the population of residual disseminated tumor cells (DTCs) is poorly understood. Large studies have shown the presence of DTCs in bone marrow (BM) to be a strong predictor of recurrence over the next 5 years [3, 4]. However, about 60% of the DTC-positive patients remained relapse-free until the end of the follow-up period. Consequently, there is an urgent need for markers to disclose the functional state of DTCs and evaluate their progression potential. Such markers may help us to understand the biology of dormant DTCs in patients and as decision-making tools for current and new therapies.

Our experimental model studies of DTC dormancy revealed that NR2F1, an orphan nuclear receptor of the retinoic acid receptor family, is commonly downregulated in human cancer and metastatic tissues [5–7]. In contrast, in a PDX model of squamous carcinoma, NR2F1 was upregulated in the DTCs that entered spontaneous dormancy [6], and additional results suggested that NR2F1 may pinpoint dormant DTCs in different cancer types [6]. DTC analysis in the experimental models indicated that when 40–50% of DTCs displayed nuclear NR2F1, this correlated with quiescence markers, other dormancy markers such as DEC2 and SOX9, and lack of proliferation [6, 8]. In addition, a frequency of less than 20% of DTCs positive for NR2F1 correlated with a lack of expression of the above markers of dormancy, quiescence, and proliferation. Metastatic and local recurrence samples in head and neck squamous cell carcinoma that clearly escaped dormancy showed less than 5% of tumor cells positive for NR2F1 (supplementing data in [6]). Furthermore, dormant DTCs upregulated genes linked to NR2F1 signaling, including several retinoic acid-regulated genes [6]. In prostate cancer samples, we found that 43–47% of DTCs from patients with no evidence of disease after many years of relapse-free follow-up showed NR2F1 mRNA upregulation, compared with 10% of the DTCs in advanced prostate cancer [6]. Altogether, these results support further testing of NR2F1 as a dormancy marker in solitary DTCs from clinical samples, including assessment of cut-off values to classify patients according to NR2F1 expression.

Three Norwegian early breast cancer cohorts were previously analyzed for DTCs in the BM [9–14] using the standard immunocytochemical method (standard ICC) [15, 16]. Clinical follow-up identified the presence of DTCs to be a significant, independent predictor of unfavorable outcome [9–12]. To explore the functional state of the DTCs, we optimized double immunofluorescence (DIF) protocols for detection of NR2F1 and Ki67 on DTCs and analyzed selected BM samples from these three breast cancer cohorts with comparison to clinical parameters. Our study is the first to translate findings from basic biological mechanism analysis of DTC dormancy to the clinical situation.

## Materials and methods

### Breast cancer patient cohorts

**Fig. 1:**
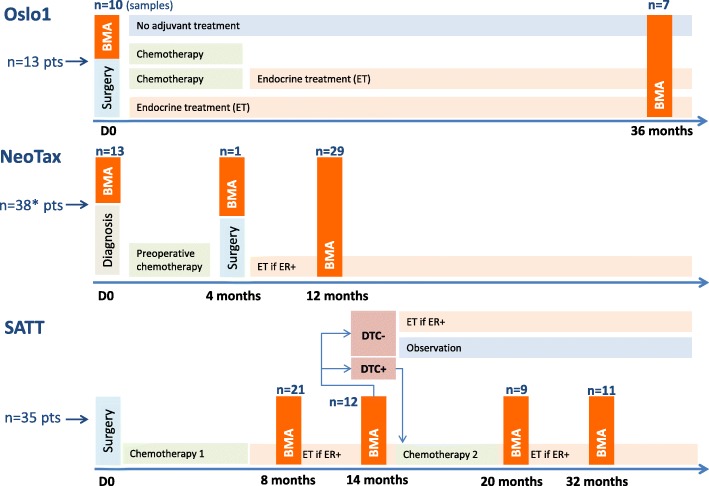
Clinical studies overview. Overview of the clinical studies, number of patients, and number of samples analyzed by DIF in the present study. Bone marrow aspiration (BMA) time points are indicated, as well as therapy administered. *One patient had a BMA performed at an unknown time point; however not harboring any disseminated tumor cells (DTC) by DIF. ER estrogen receptor

The patient material includes cytospins with BM mononuclear cells (MNCs) from breast cancer patients included in one of three different Norwegian studies in the period from 1995 to 2008. An overview of the studies and included patients is presented in Fig. 1, and below.

The NeoTax study enrolled 260 patients with stage III/IV breast cancer between 1997 and 2003 and randomly allocated them to treatment with paclitaxel or epirubicin, with a crossover between treatment arms if there was no response [9, 13, 14]. Stage IV patients were included only if they harbored a locally advanced disease (T3/T4 and/or N2/N3) with limited distant metastases. After chemotherapy, mastectomy with axillary clearance was performed, followed by radiotherapy and antihormonal therapy when estrogen receptor (ER)-positive. BM aspirations for DTC analysis were performed prior to the start of chemotherapy (BM1), at surgery (BM2), and 12 months after randomization (BM3).

The Oslo1 observational study enrolled 920 patients with stage I/II breast cancer between 1995 and 1998, and submitted them to standard adjuvant therapy, antihormonal therapy, and radiotherapy according to Norwegian guidelines at the time of the study. BM aspirations for DTC analysis were performed at surgery (BM1), and after 3 years of follow-up (for about one-third of the patients; BM2) [10, 11].

The SATT study [12, 17] enrolled 1121 patients with operable breast cancer between 2003 and 2008. In addition to chemotherapy, patients received antihormonal therapy, radiotherapy, and from 2005 also herceptin if HER2-positive, according to Norwegian guidelines at the time of the study. Patients who had completed six cycles of standard adjuvant fluorouracil, epirubicin, and cyclophosphamide (FEC) chemotherapy underwent BM aspiration 2 to 3 months (BM1) and 8 to 9 months (BM2) after FEC. The presence of DTCs in BM was determined by immunocytochemistry. If one or more DTCs were present at BM2, six cycles of docetaxel (100 mg/m^2^, once every 3 weeks) were administered, followed by DTC analysis 1 and 13 months after the last docetaxel infusion (BM3 and BM4).

### Preparation of bone marrow mononuclear cell samples and cytospins

Bone marrow was aspirated in heparin (1000 IE/ml; 0.5 ml per 10 ml BM) from iliac crests bilaterally under local anesthesia (5–10 ml per site) and pooled into one tube. The samples were stored at room temperature until processing within 24 h. The aspirates were diluted 1:1 in phosphate-buffered saline (PBS; Gibco, Life Technologies) and separated by density centrifugation using Lymphoprep (Axis-Shield, Oslo, Norway). Mononuclear cells were collected from the interphase layer, washed in 1% fetal calf serum in PBS (Gibco), and resuspended to 1 × 10^6^ cells/mL. Cytospins were prepared by centrifugation of the BM MNCs down to poly-l-lysine-coated glass slides (5 × 10^5^ MNCs/slide) in a Hettich cytocentrifuge (Tutlingen, Germany), air-dried at room temperature overnight, and stored at −80 °C until immunostaining.

### Patient material

For all the studies, the large majority of DTC-positive samples had only one detectable DTC in the original standard ICC analysis. A minority of the original samples contained ≥ 2–5 up to several thousand DTCs. For the present study, stored frozen cytospins prepared in parallel to the cytospins used for the initial (original) analysis were used when available. For some samples, viable BM MNCs stored in liquid nitrogen were thawed and new cytospins were prepared. To increase the chance of detecting DTCs in the study samples, we primarily selected patients having ≥ 3 DTCs per 2 × 10^6^ BM MNCs by the original DTC analysis. However, patients with 0–2 DTCs were also included. When available, we prioritized BM from patients where successive samples over time were available. For some patients, no more spare BM was available for the present analysis. Normally, two cytospins containing in total 1 × 10^6^ BM MNCs with adequate staining quality were analyzed for NR2F1 and for Ki67. The staining of samples and scoring of Ki67 and NR2F1 were performed by EB and MCR without access to the clinical database for the trials or information about time to relapse.

Based on this, the present study included cytospins of BM MNCs from a total of 86 patients categorized as DTC-positive by the original “gold standard” DTC analysis performed prospectively within the original studies (DTC-positivity in at least one original sample if more than one BM aspiration was performed; ICC APAAP technique, four cytospins, 2 × 10^6^ BM MNCs analyzed) [15, 16], of which 13 were included in Oslo1, 38 in the NeoTax study, and 35 in the SATT study (Fig. 1). For 24 of these patients, successive analyses from two or three time points were analyzed (20 at two time points and 4 at three time points). Samples from 11 patients with no detectable DTCs by the original DTC analysis were also included. In total, 127 samples were analyzed (all presented in Additional file 1: Table S1).

### NR2F1/Ki67 and AE1AE3 double immunofluorescence staining protocol

Double immunofluorescence was performed using the broad-specter anticytokeratin (anti-CK) monoclonal antibodies (mAbs) AE1/AE3 combined with anti-COUP TF1/NR2F1 for expression of dormancy. From a selection of the samples, parallel (additional) cytospins were available and DIF was performed using anti-CK mAbs AE1/AE3 combined with the marker Ki67 for proliferation expression. Cytospins (0.5 × 10^6^ MNCs/slide) were fixed for 12 min in methanol/acetone (1:1) at room temperature and briefly air-dried, permeabilized in Triton X-100 (0.1% in PBS (DPBS Gibco-CaCl_2_/MgCl_2_)) for 7 min, followed by a wash in PBS. The slides were then incubated for 45 min with one of the following mAbs: NR2F1 Anti-COUP TF1 (Abcam Ab 41,858; 10 μg/mL) or anti-Ki67 clone MIB-1 (DAKO M7240; 1.15 μg/mL). They were subsequently labelled with Alexa Fluor 488 goat anti-mouse IgG (H + L) (Molecular Probes 11029; 4 μg/mL) and incubated for 45 min. To block for cross-reactions (Ki67) the slides were then incubated with a mouse mAb MOPC21 (Sigma-Aldrich M9269; 20 μg/mL) for 20 min. Finally, a combination of the two anti-CK mAbs AE1 and AE3 (Chemicon Millipore mAbs 1611/1612) were added, fluorescently labelled by Zenon 555 (2 μg of the AE1/AE3 combination per slide was labeled by the Zenon 555 mouse IgG labeling kit (Life technologies Molecular Probes, Z25005) diluted to 20 μg/mL), and the slides were incubated for 45 min. Slides were washed 2 × 5 min in PBS, then sealed with ProLong Gold antifade reagent with DAPI (Life technologies P36931) and cover-slipped. Throughout the protocol, the slides were washed 2 × 5 min in PBS after each antibody incubation step. All antibodies were diluted in PBS/ 0.5% Tween20/ 5% normal goat serum. Cytospins (0.5 × 10^6^ MNCs/slide) spiked with 1% MCF7 or SKBR3 breast cancer cell line cells were used as positive controls for the anti-CK staining and for optimization of the DIF protocols. Among the normal BM cells in the patient cytospins there were both Ki67-positive cells and cells with 0–5 small NR2F1 signals (see a more detailed description below), serving as internal positive controls for the NR2F1 staining.

### Scoring of individual DTCs and NR2F1/Ki67 expression

Stained cells were identified by manual screening in a Leica Microsystems DMI6000B fluorescence microscope, using 20×, 40×, and 63× objectives. Only AE1/AE3-positive cells with a morphology compatible with tumor cells were scored as DTCs [15, 16].

**Fig. 2:**
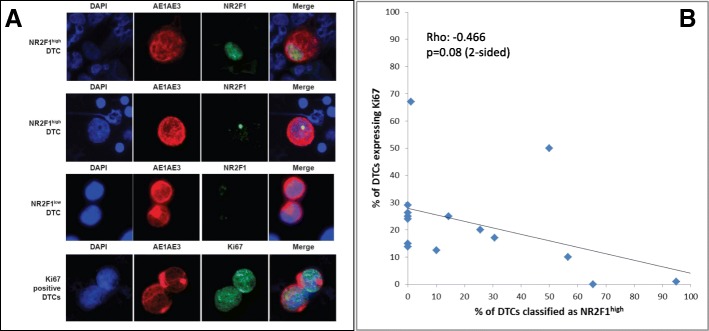
Images of disseminated tumor cells (DTCs) stained by double immunofluorescence (AE1AE3/NR2F1 and AE1AE3/Ki67) and correlation between Ki67 and NR2F1 expression. **a** DTCs from the BM of study patients analyzed by DIF. The strong and irregular cytoplasmic cytokeratin staining (AE1AE3 antibody, in red fluorescence) identifies these cells as DTCs among the normal BM MNCs (AE1AE3-negative). The two upper rows show NR2F1^high^ DTCs with the presence of nuclear NR2F1 signal clusters (in green fluorescence; first row) or one large size NR2F1 signal (i.e., larger than the size range observed in normal BM MNCs; second row). Third row shows two DTCs classified as NR2F1^low^ containing only two (lower cell) or three (upper cell) small NR2F1 signals, i.e., expression not exceeding what may be observed in normal BM MNCs. The bottom row shows two DTCs positive for Ki67 (in green). **b** Comparison of the expression on DTC of NR2F1 versus the proliferation marker Ki67. Results from DIF analysis of Ki67/AE1AE3 versus NR2F1/AE1AE3, respectively, on 15 of the BM samples presented in Table 1, where additional cytospins were available for both analyses

The definitions of DTC as either NR2F1^low^ or NR2F1^high^ used in this study were determined prior to starting the screening of the patient samples. When optimizing the AE1AE3/NR2F1 DIF protocol we observed that a large majority (> 99%) of normal blood and BM MNCs showed from zero up to two small NR2F1 nuclear localization signals (Additional file 2: Figure S1), and only occasionally did normal BM cells harbor up to five small signals. In contrast, in MCF7 and SKBR3 breast cancer cell line cells, and in test patient samples harboring many DTCs, a range from zero up to many, often large, irregular NR2F1 nuclear localization signals were observed, often with an appearance compatible with localization in the nucleoli (Additional file 2: Figure S1); in some DTCs cytoplasmic signals could also be observed. From our previous immunofluorescence experience in experimental models and cell lines in vivo [6, 8] we have defined NR2F1^high^ cells as cells with a strong NR2F1 signal detected in all the nuclear area (Fig. 2a, first row) or deposited as dotted or large irregular nucleolar-like signals (Fig. 2a, second row). In proliferative human and experimental tumors, the NR2F1 signal is either negative (no signal at all) or a weak speckled signal, except in certain areas that are hypoxic [8]. Based on these data from both experimental studies and testing on MCF7 and SKBR3-spiked normal MNCs, we defined as NR2F1^low^a range of NR2F1 immunostaining from entirely negative up to five small signals as seen in normal MNCs (Additional files 2 and 3: Figures S1 and S2). A NR2F1 staining > 5 small signals and/or large signals (≥ 1), and/or the presence of signal clusters defined DTCs as NR2F1^high^ (Fig. 2, Additional files 2 and 7: Figures S1 and S2).

In accordance with the preclinical study data presented above, we chose to classify, a priori, samples with ≥ 50% NR2F1^high^ DTCs as “dormant” and samples with < 50% NR2F1^high^ DTCs as “non-dormant”.

One of the patients with DTC-negative status according to the original DTC analysis had one detectable DTC by the DIF analysis and was not classified according to “dormant” versus “non-dormant” status.

A Ki67-expressing DTC was defined as a cell exhibiting nuclear immunostaining of Ki67.

### Statistics

The association between DTC status/characteristics and distant disease-free interval (DFI) was analyzed. Distant DFI was defined as survival without distant breast cancer recurrence or breast cancer death, and was constructed using Kaplan-Meier curves with accompanied *P* values obtained from a log-rank test. SPSS software was used for statistical analysis.

## Results

Bone marrow cytospins from 86 DTC-positive patients identified by the original DTC staining procedure were analyzed by DIF for cytokeratin (AE1/AE3) and NR2F1 expression as described in Materials and methods. An overview of the BM aspiration (BMA) time points for the included patients is presented in Fig. 1. From 24 of these patients, BM samples at ≥ 2 time points were available for DIF analysis.

Cytokeratin-positive cells were classified as either NR2F1^high^ or NR2F1^low^ according to the level and pattern of expression (see Materials and methods, Fig. 2, and Additional files 2 and 3: Figures S1 and S2).

Expression of Ki67 was analyzed in parallel with the NR2F1 analyses on additional available cytospins from 15 DTC-positive patients (Table 1 and Fig. 2b). Of the total 103 samples found to be DTC-positive (i.e., ≥ 1 detectable DTC) by the original DTC staining procedure (of 2 × 10^6^ BM MNCs), 32 (31%) had cytokeratin-detectable DTCs in the DIF analysis (of 1 × 10^6^ BM MNCs) (Additional file 4: Table S2), in accordance with an expected lower sensitivity of this analysis. Twenty-four samples submitted to DIF had been concluded as DTC-negative by the original DTC analysis. These included 13 samples from 11 patients with no original detectable DTCs. One of the samples had one detectable DTC by DIF, and the remaining 23 were DTC-negative (Additional file 4: Table S2). Data on the original DTC-positive patients with DIF-positive results and available clinicopathological characteristics are presented in Table 1.

**Table 1:** Overview of all originally disseminated tumor cell (DTC)-positive patients with detectable DTCs by double immunofluorescence technique and clinicopathological parameters

Patient identifier	Study	BMA time points^a^	NR2F1/AE1AE3 DIF analysis		Ki67/AE1AE3 DIF analysis		HR	HER2	T status	N status	Time (months) from BMA to systemic relapse or BC death	Relapse status or BC death (if not recorded relapse)	Time (months) from BMA to last observation if no relapse	Comment
			No. of DTCs	% NR2F1^high^ DTCs	No. of DTCs	% Ki67+ DTCs								
20	N	(BM1)-**BM3**	35	0	7	28.6	pos	nd	3	2	0.30	Bone and visceral		
74	S	(BM1-BM3)-**BM4**	3	0	nd	nd	pos	neg	1	1	1.09	Bone		
5	O	(BM1)/**BM2**	1000	0	1000	15.0	pos	pos	1	1	1.15	Bone		
60	S	(BM2)-**BM3**	1000	0^b^	84	26,2	pos	neg	2	1	1.84	Bone		
27	N	(BM1)-**BM2**	5	0^c^	nd	nd	neg	nd	3	0	5.69	Visceral		
62	S	**BM2**	40,000	0	nd	nd	pos	neg	2	0	5.79	Bone and visceral		Chemo after BMA
4	O	(BM1)-**BM2**	9	0	23	21.7	pos	neg	3	1	7.50	Bone and visceral		
3	O	**BM1**	11	0	nd	nd	pos	neg	2	1	11.22	Visceral		
78	S	(BM1)-**BM2**-(BM3)	26	0	29	13.8	pos	neg	2	3	13.19	Bone and visceral		Chemo after BMA
13	O	**BM1**	79	0	nd	Nd	neg	pos	1	1	13.22	Bone		Chemo after BMA
17	N	**BM3**	5	0	4	25.0	pos	nd	3	1	17.57	Bone and visceral		
34	N	**BM1**-(BM2)	2	0	nd	nd	pos	neg	3	1	47.43	Visceral		Chemo after BMA
12	O	**BM2**	16	0	nd	nd	pos	neg	2	1		No relapse	57.73	
66	S	**BM1**-(BM4)	3	0	nd	nd	pos	neg	1	2		No relapse	87.50	Chemo after BMA
11	O	(BM1)-**BM2**	500	1.0	87	66.7	neg	pos	3	1	3.72	BC death		
23	N	**BM3**	50	10.0	16	12.5	pos	pos	3	0	13.55	BC death		
35	N	(BM1)-**BM3**	7	14.3	12	25.0	pos	nd	3	2	N/A	^d^		Met. before BMA
48		(BM1)-**BM3**	17	25.5	10	20.0	pos	nd	3	0	12.24	Bone		
41	N	**BM3**	111	30.6	82	17.1	pos	nd	4	1	N/A	^d^		Met. before BMA
36	N	(BM1)-**BM3**	6	50,0	4	50.0	pos	nd	3	1	25.33	Bone		
84	S	(BM1)-**BM2**	2	50.0	nd	nd	pos	neg	2	2		No relapse	55.53	Chemo after BMA
9	O	**BM1**	48	56.3	nd	Nd	neg	neg	2	1	N/A	Bone^d^		Met. at BMA
85	S	(BM1)-**BM2**	106	56.6	70	10.0	pos	neg	2	2		No relapse	56.02	Chemo after BMA
69	S	(BM1)-**BM2-**(BM3)	52	65.4	35	0	pos	neg	2	2	15.10	Visceral		Chemo after BMA
6	O	(BM1)-**BM2**	233	94.9	400	1.0	neg	pos	1	1	N/A	Visceral		Met. before BMA
57	S	(BM3)-**BM4**	6	100	nd	nd	neg	nd	2	0		No relapse	59.93	

*BC* breast cancer, *Chemo* chemotherapy, *DIF* double immunofluorescence, *HR* hormone receptor, *Met*. metastasis, *N* Neotax, *N/A* not applicable, *nd* not determined, *neg* negative, *O* Oslo1, *pos* positive, *S* SATT

**Fig. 3:**
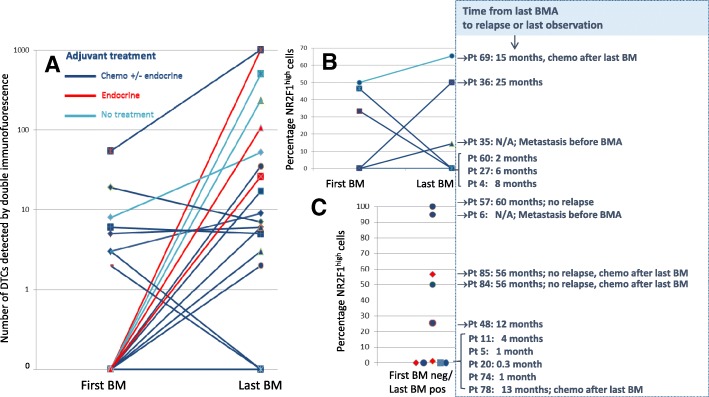
Disseminated tumor cell (DTC) status by DIF and NR2F1 expression in patients with bone marrow (BM) samples available at two time points. Results of AE1AE3/NR2F1 DIF analysis performed on 24 patients classified as DTC-positive in the original DTC analysis, and with available BM samples from two aspiration time points. The number of DIF cytokeratin-positive DTCs (**a**), the proportion of NR2F1^high^ DTCs in patients with DIF DTC-positive status at both BM aspiration (BMA) time points (**b**), and the proportion of NR2F1^high^ DTCs in patients with DIF DTC-positive status in the second but not the first BMA (**c**) are presented. The right sections of **b** and **c** show time to relapse or last observation and additional clinical information for the patients presented in **b** and **c**. Chemo chemotherapy, N/A not applicable, neg negative, pos positive, Pt patient

^a^The bone marrow aspiration (BMA) time points for each patient are noted. In this table, the DTC results are only presented for the BMAs highlighted in bold (last positive sample); results from the other BMA time points are available in Additional file 1 (Table S1) and partly in Fig 3

^b^In the first bone marrow (BM) sample, 46.3% of the DTCs were NR2F1^high^

^c^In the first BM sample, 33.3% of the DTCs were NR2F1^high^

^d^Metastasis before BMA

The DIF analysis revealed that most of the analyzed patients (24 out of 26) with CK-positive DTCs had ≥ 3 detectable cells and 16 had ≥ 10 DTCs in at least one BM sample, representing a patient group with high risk of metastasis (Table 1, Additional file 5: Table S3). Indeed, 81% of the DTC-positive patients developed metastasis after the BMA (*n* = 17) or had metastasis at time of the BM aspiration (*n* = 4) (Table 1). Half (*n* = 13) of the patients had > 1% of NR2F1^high^ DTCs in at least one BM sample and 26.9% (*n* = 7) had ≥ 50% NR2F1^high^ DTCs. The latter parameter (≥ 50% NR2F1^high^ DTCs) was chosen as the a priori cut-off for classifying the patient as having a “dormant profile” in accordance with previous experimental studies [6, 8]. Of the samples with detectable NR2F1^high^ DTCs, the median proportion of NR2F1^high^ DTCs was 50%.

To explore changes in the expression of NR2F1 over time and during treatment, DIF analysis was performed on the 24 cases classified as DTC-positive in the original DTC staining procedure and with available samples from BM aspiration at two time points (see Additional file 6: Figure S3 for the original DTC staining results). Of the cases analyzed, 16 received chemotherapy (± endocrine treatment), 5 endocrine treatment only, and 3 no systemic treatment between the BM aspirations (Additional file 7: Table S4). The number of DIF-detected CK-positive DTCs and proportion of NR2F1^high^ DTCs are presented in Fig. 3. The results showed different patterns of change and did not appear to be related to the type of adjuvant treatment (Fig. 3). Three of the six patients with ≥ 50% NR2F1^high^ DTCs at the last BM analysis did not experience relapse. In contrast, 7 of 8 patients with ≤ 1% NR2F1^high^ DTCs at the last BM analysis had systemic relapse or breast cancer death within 12 months (i.e., < 8 months) (Fig. 3b, c). Additional information on the original DTC status, NR2F1 expression, and Ki67 expression of these patients are presented in Additional file 8 (Table S5). All patients with systemic relapse or breast cancer death within 12 months had ≤ 1% NR2F1^high^ DTCs (Table 1).

**Fig. 4:**
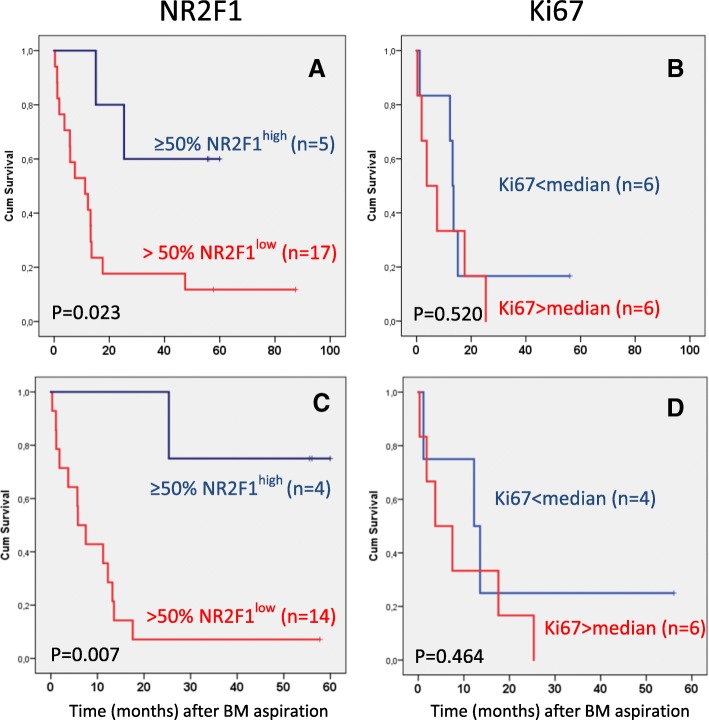
Survival analyses in relation to DTC dormancy profile and Ki67 status. Survival analyses (time to systemic relapse/breast cancer death) in relation to NR2F1 (**a**,**c**) and Ki67 profile (**b**,**d**) of DTCs (at last DIF DTC-positive bone marrow (BM) aspiration). **a**,**b** Patients being nonmetastatic at last DIF DTC-positive BMA. **c**,**d** Patients being  nonmetastatic at last DIF DTC-positive BMA with no subsequent BM analysis performed. Cum cumulative

Table 2 presents the systemic relapse status among the patients with 1 and ≥ 2 BM aspiration time points in combination, according to the proportion of NR2F1-expressing cells in the DTC-positive cases (last positive BM aspiration time point if > 1 performed). Of the patients with predominantly NR2F1^low^ DTCs, 90% had, or  experienced, systemic relapse or breast cancer death and 67% were recorded with bone metastasis. Similar figures were observed for those with ≤ 1 NR2F1^high^ DTCs. In contrast, in those patients with ≥ 50% NR2F1^high^ expressing DTCs, 57% had, or experienced, systemic relapse and 29% were recorded with bone metastasis. Survival analysis of all nonmetastatic patients at the time of last DIF DTC-positive BM aspiration revealed a difference in distant DFI (Fig. 4a; *p* = 0.023). Excluding patients analyzed for DIF-positive DTCs (with a negative result) at a subsequent BMA time point (*n* = 18), 93% experienced systemic relapse/breast cancer death and 75% bone metastasis among the patients with a “non-dormant” DTC classification. One of the four patients with ≥ 50% NR2F1^high^ DTCs (a “dormant” DTC classification) experienced bone metastasis. Analysis of distant DFI among these 18 patients indicated a survival difference between the patients classified by DTCs as having < 50% versus ≥ 50% NR2F1^high^ expressing cells (Fig. 4c; *p* = 0.007). A few patients had exceptionally high DTC numbers. A survival analysis without the patients with ≥ 500 DTCs gave similar results (*p* = 0.014; Additional file 9: Figure S4A). The patients included in the NeoTax study had higher stages (all with locally advanced disease) than the two other cohorts. Excluding these patients from the survival analysis did not change the results (*p* = 0.022; Additional file 9: Figure S4B).

**Table 2:** NR2F1 expression and clinical outcome

	Fraction of DTCs categorized as NR2F1^high^		Distant metastasis (all) or death from breast cancer (%)	Bone metastasis^b^ (%)
All patients^a^ (*n* = 26)	< 50%	0 to < 50% NR2F1^high^	17/19 (89.5)	10/15 (66.7)
		0–1% NR2F1^high^	13/15 (86.7)	9/14 (64.3)
	50–100%		4/7 (57.1)	2/7 (28.6)
Patients without metastasis prior to last DTC-positive BMA and no negative DTC status at subsequent BMA (*n* = 18)	< 50%	0 to < 50% NR2F1^high^	13/14 (92.9)	9/12 (75.0)
		0–1% NR2F1^high^	11/12 (91.7)	8/11 (72.7)
	50–100%		1/4 (25.0)	1/4 (25.0)
Patients with no metastasis at time point for last DTC-positive BMA, no negative DTC status at subsequent BMA, and no chemotherapy after the BM analysis (*n* = 14)	< 50%	0 to < 50% NR2F1^high^	11/12 (91.7)	7/10 (70.0)
		0–1% NR2F1^high^	9/10 (90.0)	6/9 (66.7)
	50–100%		1/2 (50.0)	1/2 (50.0)

If analysis was performed at more than one time point, the last disseminated tumor cell (DTC)-positive sample is included

*BMA* bone marrow aspirate

^a^Includes results from 4 patients with metastases detected before bone marrow (BM) analysis and 8 patients receiving chemotherapy after the BM analysis

^b^No information on bone metastasis status was available from four patients in total

Limiting the analysis to only those with no chemotherapy after the last BMA revealed similar results, although the interpretation is restricted by the low number of patients with ≥ 50% NR2F1^high^ DTCs (*n* = 2) (Table 2 and Additional file 10: Figure S5; *p* = 0.091).

To compare the expression of NR2F1 and the proliferation marker Ki67, 1–2 additional cytospins from 15 of the BM samples presented in Table 1 were analyzed by Ki67/pan-cytokeratin DIF. NR2F1 and Ki67 expression were not examined in the same DTCs (cytospins), and therefore the combined expression pattern at the single DTC level could not be addressed. The results showed that the proportion of Ki67-positive DTCs was weakly negatively correlated with the proportion of NR2F1^high^ DTCs (ρ = −0.466; *p* = 0.08; Fig. 2b), bearing in mind the low number of cases analyzed for both Ki67 and NR2F1. Survival was not different for patients classified into subgroups by Ki67 expression in DTCs using median dichotomization (Fig. 4b, *p* = 0.520; Fig. 4d, *p* = 0.464), or by the same cut-off value as for NR2F1 (*p* = 0.753, data not shown). The survival difference between patients with NR2F1^high^ and NR2F1^low^ expressing DTCs were similar if the analysis was restricted to only the patients with samples analyzed for Ki67 (Additional file 11: Figure S6; *p* = 0.019 and *p* = 0.026 for the same patient categories as presented in Fig. 4).

## Discussion

To further improve curative treatment of breast cancer, we need to identify patients with MRD and characterize the potential for MRD progression. To the best of our knowledge, this is the first report exploring dormancy marker profiling in DTCs in breast cancer patients. The analysis of NR2F1 expression, a critical node in tumor dormancy induction, can potentially differentiate between active occult tumor cells giving a risk for early metastasis development and more long-term quiescent DTCs. Such information may potentially contribute to future clinical decisions based on minimal residual cancer detection and its state of activation.

We observed that the samples from patients with very early systemic relapse (within 12 months) carried only NR2F1^low^ (non-dormant) DTCs in the last BM sample (≤ 1% NR2F1^high^ DTCs). This included patients that transitioned from having NR2F1^high^ expressing DTCs to a NR2F1^low^ DTC state in consecutive samples (Fig. 3). Likewise, longer disease-free interval/no detectable metastases were indicated among patients with a presence of predominantly NR2F1^high^ DTCs. This was further supported by the result from survival analysis of nonmetastatic patients showing a difference in metastasis-free interval in subgroups according to NR2F1^high^ expression (Fig. 4a). However, the results should be interpreted with caution due to the restricted number of patients analyzed and the heterogeneity in patient population and treatment. Nevertheless, the data provide clinical support to the abundant previous experimental and some clinical data (mRNA measurements) identifying NR2F1 as a candidate marker for clinically relevant characterization of MRD [6], and that NR2F1 may serve to identify DTC long-term dormancy candidates even among patients harboring larger number of DTCs. Indeed, the patients studied were not selected to be obvious DTC dormancy candidates by a long (many years) relapse-free follow-up period. Most of the BM samples were collected ≤ 3 years after diagnosis and were enriched for cases with ≥ 5 DTCs, a known poor prognostic feature [3, 4, 9–12]. Moreover, although the DTC Ki67 expression showed a weak negative correlation with NR2F1^high^ DTCs (Fig. 2b), no significant association with clinical outcome was observed. This indicates that a proliferation marker such as Ki67 is insufficient to characterize the MRD cell population.

Since Ki67 detects all phases of the cell cycle, except G0, it is possible that it may not accurately pinpoint true dormant cells. In our experience, retinoblastoma protein (pRb) and P-H3-negative, p27-positive cells are better indicators of a quiescent NR2F1-positive DTC [6, 8, 18, 19]. The Ki67 result may place into the proliferative population bin, cells that are in a G0/G1 boundary and arrested or slow cycling. Furthermore, it may classify nonarrested cells transiting through G0 as nonproliferative. In contrast, NR2F1 expression is remarkably stable, epigenetically controlled, and associated with a repressive chromatin state observed in terminally senescent or differentiated cells [6]. These data suggest that NR2F1 marks a durable, more long-lived phenotype of growth arrest. The presented data and results from our experimental models also suggest that NR2F1 is associated with cellular dormancy (quiescence) and not tumor mass dormancy (representing a small cancer cell mass that cannot surpass a certain size) characterized by a balance between proliferation and apoptosis where arrest is never observed. [20]. In our experience and that of other investigators, the latter phenomenon is not observed in solitary DTC dormancy [6, 8, 18, 19, 21, 22]. Published data also suggest that the mechanisms driving solitary DTCs share a significant overlap with those regulating adult stem cell quiescence [6, 20, 23], which is a cellular dormancy mechanism. These mechanisms may explain the divergence between Ki67 and NR2F1, although additional validation is needed because these markers were not analyzed for in the same DTCs. Nevertheless, results presented in this study and prior results strongly support the concept [20] that lack of proliferation is not the same as dormancy, but rather that proliferative arrest is one characteristic of the dormancy program. This underpins the need for markers that can identify the biological key mechanisms for dormancy-associated quiescence that are different from the absence of cell cycle phase markers.

Improved techniques to assess the MRD population and their dormant or reactivating state will be key to identifying the risk of future metastasis despite undergoing standard treatment. This opens the way for testing new treatments that prevent metastasis by inducing/enforcing dormancy, and/or to eradicate MRD [2, 6]. Dormant cancer cells can evade chemotherapy and also express pluripotency genes that keep them in a long-term reawakening probability state [6]. Retinoic acid and 5-azacitidine are examples of dormancy-inducing/sustaining treatment strategies, showing the ability to reprogram malignant cells into dormancy and enforce dormancy programs in already quiescent tumor cells [6, 7]. These drugs will be tested in a clinical trial of prostate cancer patients at risk of developing metastasis (Mount Sinai IRB no. 18–00226; ClinicalTrials.gov identifier, NCT03572387).

Sustaining a dormancy phenotype could have life-saving consequences. In line with this, patients with NR2F1 expression in a few DTCs appeared to have longer disease-free survival in our study. This may suggest that those few DTCs are indicative of at least two parameters that need to be further investigated: first, that residual DTCs not detected in the test clearly share the same phenotype as those detected, and second, that the test seems to also inform on patients that may have niches that are pro-dormant and thus support dormancy of the residual DTCs for longer time periods. The first possibility is supported by abundant experimental evidence for a role of NR2F1 in DTC dormancy through a microenvironmental and epigenetic program of regulation [6]. The second is less explored, but it is possible that some patients may be better producers of dormancy-inducing cues as these are commonly signals involved in adult stem cell quiescence. Furthermore, androgen deprivation treatment in prostate cancer has been linked to upregulation of NR2F1 [24], suggesting that certain commonly used therapies may induce dormancy and cooperate in a long-term response by affecting the DTCs and the host to enter a pro-dormancy state. In breast cancer, response to tamoxifen was reported to be associated with the presence of transforming growth factor (TGF)β2, a dormancy-inducing factor [25, 26]. Furthermore, a reduced androgen receptor signaling resulted in TGFβ2 upregulation in the prostate and seminal gland tissue [27]. Thus, future studies may not only focus on detection of dormant DTCs, but also investigation of whether the host is producing pro-dormancy cues.

Among all the patients included in the studies used as the source for the current project [9–12] the majority of those identified as DTC-positive had only one or two detectable DTCs across the BMA time points (based on the original analysis; NeoTax ≥ 75%, Oslo1 ≥ 87%, SATT ≥ 75%). The group of patients with such low numbers of DTCs has the most favorable survival among the DTC-positive cases [9, 28] and would also be expected to be enriched in cases with quiescent DTCs. We attempted to include both patients with originally high and low numbers of DTCs in our analysis. However, in the majority of the samples, no DTCs were detectable by DIF from patients with low DTC burden. In addition to the Poisson distribution effect, this may be for several reasons. Firstly, a reduced sensitivity of the DIF technique compared with the standard (original) APAAP ICC technique can be expected due to a stronger amplification of the signals by the APAAP (three layers) than the direct Xenon-labeling of the anti-CK antibody used in the DIF protocol. Secondly, half the number of BM MNCs (1 × 10^6^) were available for the DIF analysis. Thirdly, some of the DIF samples were prepared from liquid nitrogen-frozen MNC suspensions, which in our experience results in loss of tumor cells in some patients compared with cytospins prepared from fresh BM. Further assessment and characterization of dormancy in patients with very infrequent DTCs (i.e., below the detection level for our analysis) requires analysis of larger BM volumes in future studies, preferably using enrichment techniques [29] or automated scanning systems (http://rarecyte.com) combined with multimarker analysis. In parallel with DTC analysis, capturing functional characteristics of circulating tumor cells (CTCs) from high volumes of peripheral blood, for instance by a multitube CellSearch analysis (https://www.cellsearchctc.com/), leukapheresis-related techniques [30], or intravascular capturing devices [31, 32], would clarify whether assessment of CTCs may be used for future dormancy studies.

## Conclusions

Overall, we conclude that NR2F1 detection in BM DTCs may be a promising tool to determine the phenotype of DTCs and the prognosis of breast cancer patients. For decades, DTC biology has been relegated primarily to the area of enumeration and subsequent prognosis. Our bench-to-bedside work reveals the first potential dormancy marker that informs on the behavior of DTCs and suggests that enumeration should be followed by phenotype information. Markers such as NR2F1 coupled to DTC genetics and other host-derived indicators may provide a breakthrough in the management of MRD and metastasis prevention.

## Additional files

Additional file 1: **Table S1.** Descriptive data from all tested patients. (XLSX 15 kb)

Additional file 2: **Figure S1.** AE1AE3/NR2F1 DIF staining on normal MNCs spiked with breast cancer cell line cells. The first row shows normal MNCs (AE1AE2-negative) and one breast cancer cell line cell (MCF7; AE1AE3-positive). The MNCs contain 0–3 small/weak NR2F1 signals per nucleus and the cancer cell two similarly small signals. These cells are all defined as being NR2F1^low^ cells in the present study. Occasionally, normal BM cells harbored up to 5 small signals (not shown in the figure). The second row shows MNCs with a cluster of four breast cancer cell line cells (SKBR3), of which the lower left cell does not contain any NR2F1 signals and is therefore defined as NR2F1^low^. The third cell from the left contains 7–8 small signals, with a tendency to signal clustering, and satisfies the criteria for an NR2F1^high^ cell. In cell numbers 2 and 4 from the left, 4–5 small signals are seen. Although the signals of these cells tend to melt together in clusters/larger signals they still represent expressions below the cut-off for NR2F1^high^ classification, but are approaching the cut-off level. The cells in the second row of this figure therefore illustrate the a priori defined cut-off between NR2F1-positive and -negative cells. (NR2F1 signals in MNCs in the second row are out of focus and therefore not visible on the images). For illustration of the NR2F1 classification of DTCs within the study, see Fig. 2a and Additional file 7: Figure S2. (PDF 684 kb)

Additional file 3: **Figure S2.** Illustration of the classification system for NR2F1 expression of DTC prospectively chosen for the present study. NR2F1^low^ DTC (A–C). (A) Cluster of three DTCs identified by AE1AE3 in red fluorescence and a morphology compatible with tumor cells. Two of the DTCs have no NR2F1 signals and one has one small NR2F1 signal. Surrounding BM MNCs have 0–1 NR2F1 signals of a similar size. (B, C) One DTC with 2–3 small NR2F1 signals. Adjacent normal BM MNCs with 0–1 small NR2F1 signals. NR2F1^high^ DTC (D, E): (D) Cluster of two DTCs with coarse, partly confluent NR2F1 signals of varying sizes (signals in BM MNCs not visualized because of not being in focus). (E) Cluster of 5 DTCs, three of them defined as NR2F1^high^ because of > 5 NR2F1 signals, partly of large signal size. The remaining two DTCs, with no NR2F1 signals, are assigned NR2F1^low^, as well as the adjacent normal BM MNCs with 0–1 small NR2F1 signals. (PDF 337 kb)

Additional file 4: **Table S2.** Overview of patient material and DTC results. (DOCX 34 kb)

Additional file 5: **Table S3.** Characteristics of the DTC-positive cases by double immunofluorescence (DIF). (DOCX 33 kb)

Additional file 6: **Figure S3.** Serial BM samples: number of DTCs detected in the original DTC analysis (APAAP-ICC technique). (PPTX 128 kb)

Additional file 7: **Table S4.** Overview of received treatment between the two BM aspiration time points for the patients presented in Fig. 3. (DOCX 33 kb)

Additional file 8: **Table S5.** Additional results from the serial DTC analyses on samples presented in Fig. 3b and c (in the same order). (DOCX 39 kb)

Additional file 9: **Figure S4.** (A) Survival analyses (time to systemic relapse/breast cancer death) in relation to NR2F1 profile of DTCs for patients being nonmetastatic at the time point of last DIF DTC-positive BMA and having no subsequent BM analyzed; patients harboring ≥ 500 DTC excluded. (B) Survival analyses (time to systemic relapse/breast cancer death) in relation to NR2F1 profile of DTCs for patients being nonmetastatic at time point of last DIF DTC-positive BMA and having no subsequent BM analyzed; NeoTax study patients excluded. (PPTX 114 kb)

Additional file 10: **Figure S5.** Survival analyses according to NR2F1 and Ki67 DTC profiles of patients being nonmetastatic at the time of last DIF DTC-positive BMA, having no subsequent BM analyzed, and no chemotherapy after last BMA. (PPTX 120 kb)

Additional file 11: **Figure S6.** (A) Survival analyses (time to systemic relapse/breast cancer death) in relation to NR2F1 profile at last DIF DTC-positive BMA, restricted to those with Ki67 DTC analysis available (only patients being nonmetastatic at last DIF DTC-positive BMA included). (B) As A, but analysis restricted to patients having no subsequent BMA analyzed. (PPTX 68 kb)

## Abbreviations

APAAP: Alkaline phosphatase–anti-alkaline phosphatase
BM: Bone marrow
BMA: Bone marrow aspiration
CK: Cytokeratin
Coup TF1: Chicken ovalbumin upstream promoter transcription factor 1
CTC: Circulating tumor cell
DFI: Disease-free interval
DIF: Double immunofluorescence
DTC: Disseminated tumor cell
ER: Estrogen receptor
FEC: Fluorouracil, epirubicin, and cyclophosphamide
ICC: Immunocytochemical
mAb: Monoclonal antibody
MNC: Mononuclear cell
MRD: Minimal residual disease
NR2F1: Nuclear receptor subfamily 2, group F, member 1
PBS: Phosphate-buffered saline
pRb: Retinoblastoma protein
TGF: Transforming growth factor
