# Supplementary figures and images for: NR2F1 stratifies dormant disseminated tumor cells in breast cancer patients
Source: Breast Cancer Res. 2018 Oct 16;20:120. doi: 10.1186/s13058-018-1049-0 (PMC6190561; doi:10.1186/s13058-018-1049-0)

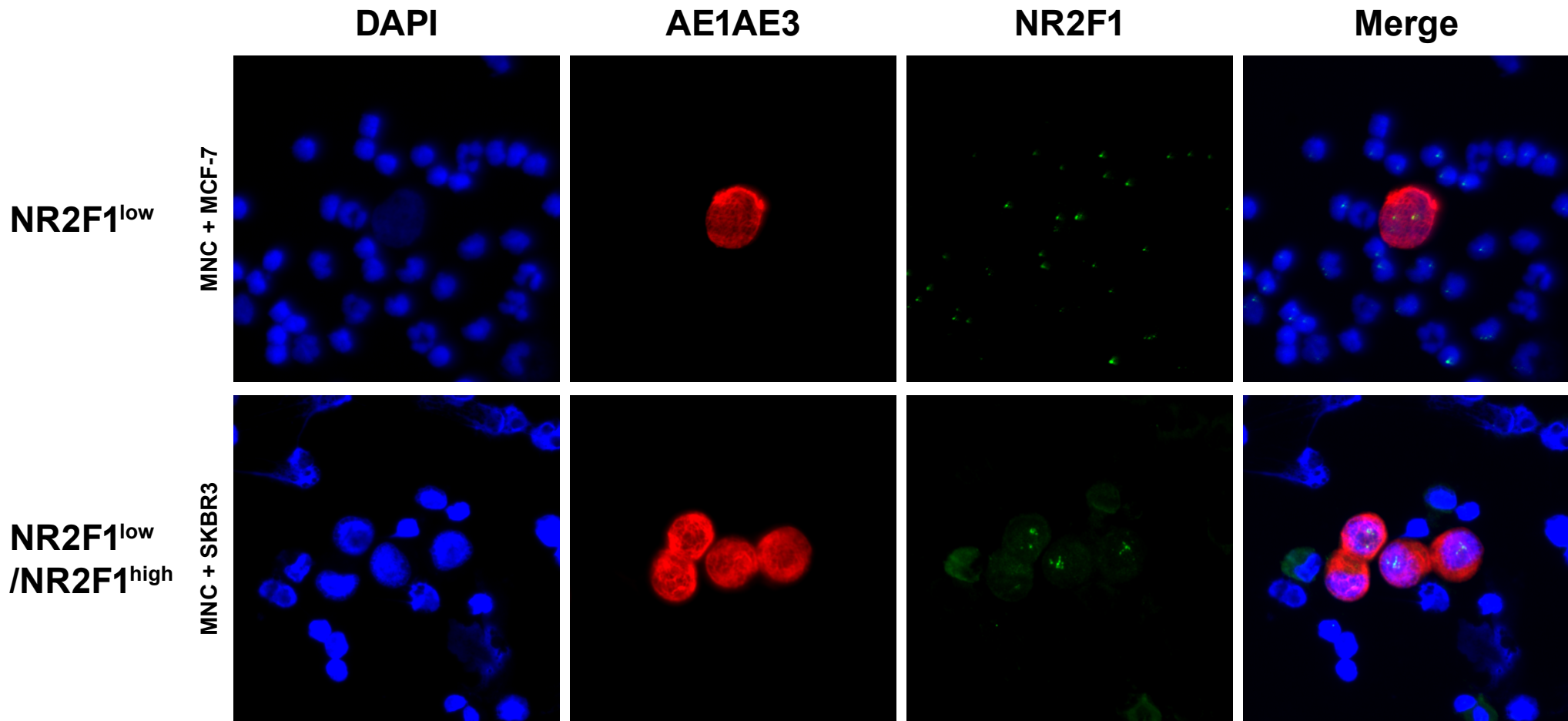

Supplement: Supplementary file 2 — Figure S1. AE1AE3/NR2F1 DIF staining on normal MNCs spiked with breast cancer cell line cells. The first row shows normal MNCs (AE1AE2-negative) and one breast cancer cell line cell (MCF7; AE1AE3-positive). The MNCs contain 0–3 small/weak NR2F1 signals per nucleus and the cancer cell two similarly small signals. These cells are all defined as being NR2F1low cells in the present study. Occasionally, normal BM cells harbored up to 5 small signals (not shown in the figure). The second row shows MNCs with a cluster of four breast cancer cell line cells (SKBR3), of which the lower left cell does not contain any NR2F1 signals and is therefore defined as NR2F1low. The third cell from the left contains 7–8 small signals, with a tendency to signal clustering, and satisfies the criteria for an NR2F1high cell. In cell numbers 2 and 4 from the left, 4–5 small signals are seen. Although the signals of these cells tend to melt together in clusters/larger signals they still represent expressions below the cut-off for NR2F1high classification, but are approaching the cut-off level. The cells in the second row of this figure therefore illustrate the a priori defined cut-off between NR2F1-positive and -negative cells. (NR2F1 signals in MNCs in the second row are out of focus and therefore not visible on the images). For illustration of the NR2F1 classification of DTCs within the study, see Fig. 2a and Additional file 7: Figure S2. (PDF 684 kb) [file 13058_2018_1049_MOESM2_ESM.pdf]

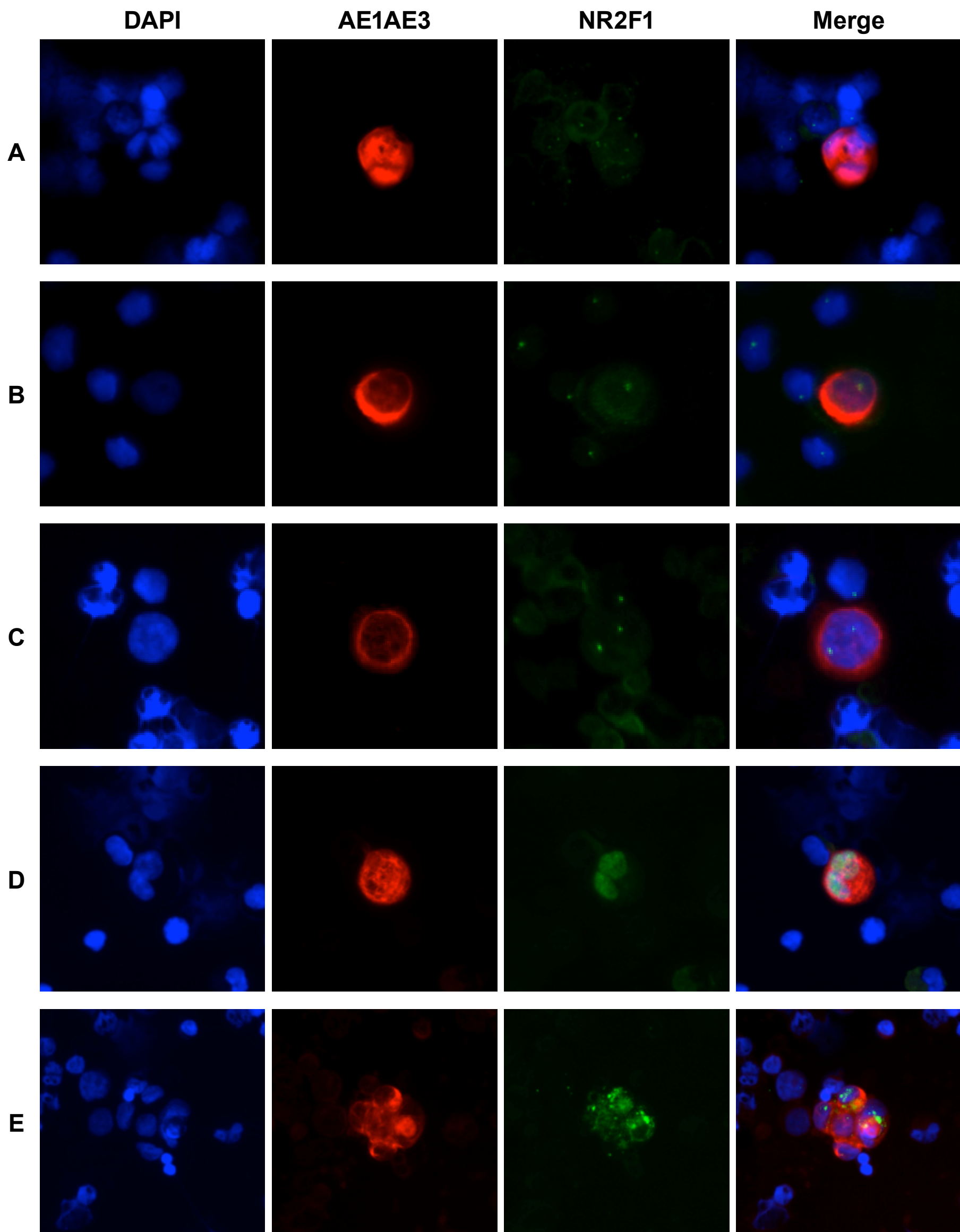

Supplement: Supplementary file 3 — Figure S2. Illustration of the classification system for NR2F1 expression of DTC prospectively chosen for the present study. NR2F1low DTC (A–C). (A) Cluster of three DTCs identified by AE1AE3 in red fluorescence and a morphology compatible with tumor cells. Two of the DTCs have no NR2F1 signals and one has one small NR2F1 signal. Surrounding BM MNCs have 0–1 NR2F1 signals of a similar size. (B, C) One DTC with 2–3 small NR2F1 signals. Adjacent normal BM MNCs with 0–1 small NR2F1 signals. NR2F1high DTC (D, E): (D) Cluster of two DTCs with coarse, partly confluent NR2F1 signals of varying sizes (signals in BM MNCs not visualized because of not being in focus). (E) Cluster of 5 DTCs, three of them defined as NR2F1high because of > 5 NR2F1 signals, partly of large signal size. The remaining two DTCs, with no NR2F1 signals, are assigned NR2F1low, as well as the adjacent normal BM MNCs with 0–1 small NR2F1 signals. (PDF 337 kb) [file 13058_2018_1049_MOESM3_ESM.pdf]

## Slide 1
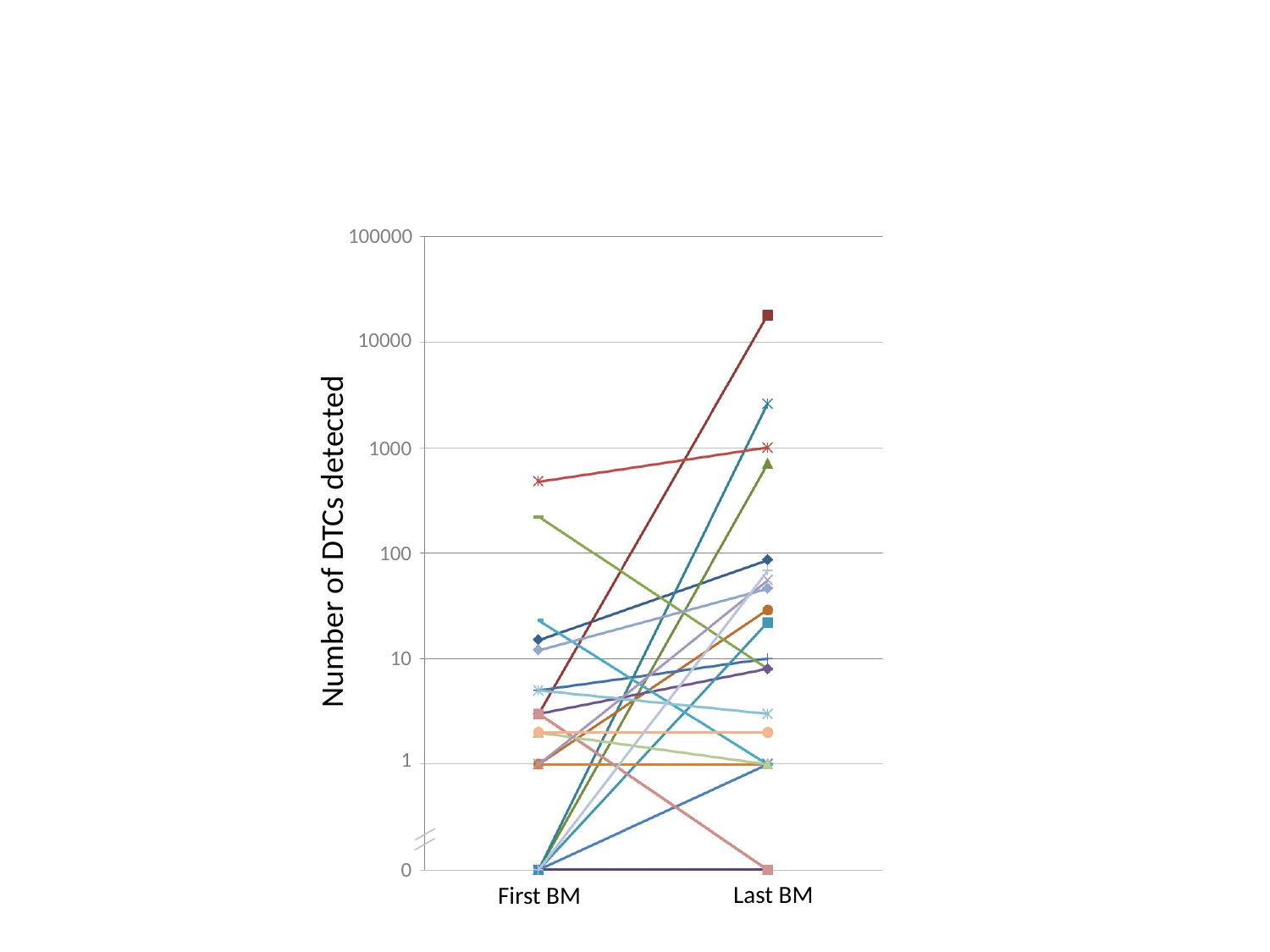

100000
10000
1000
Number of DTCs detected
100
10
1
0
Last BM
First BM

Supplement: Supplementary file 6 — Figure S3. Serial BM samples: number of DTCs detected in the original DTC analysis (APAAP-ICC technique). (PPTX 128 kb) [file 13058_2018_1049_MOESM6_ESM.pptx]

## Slide 1
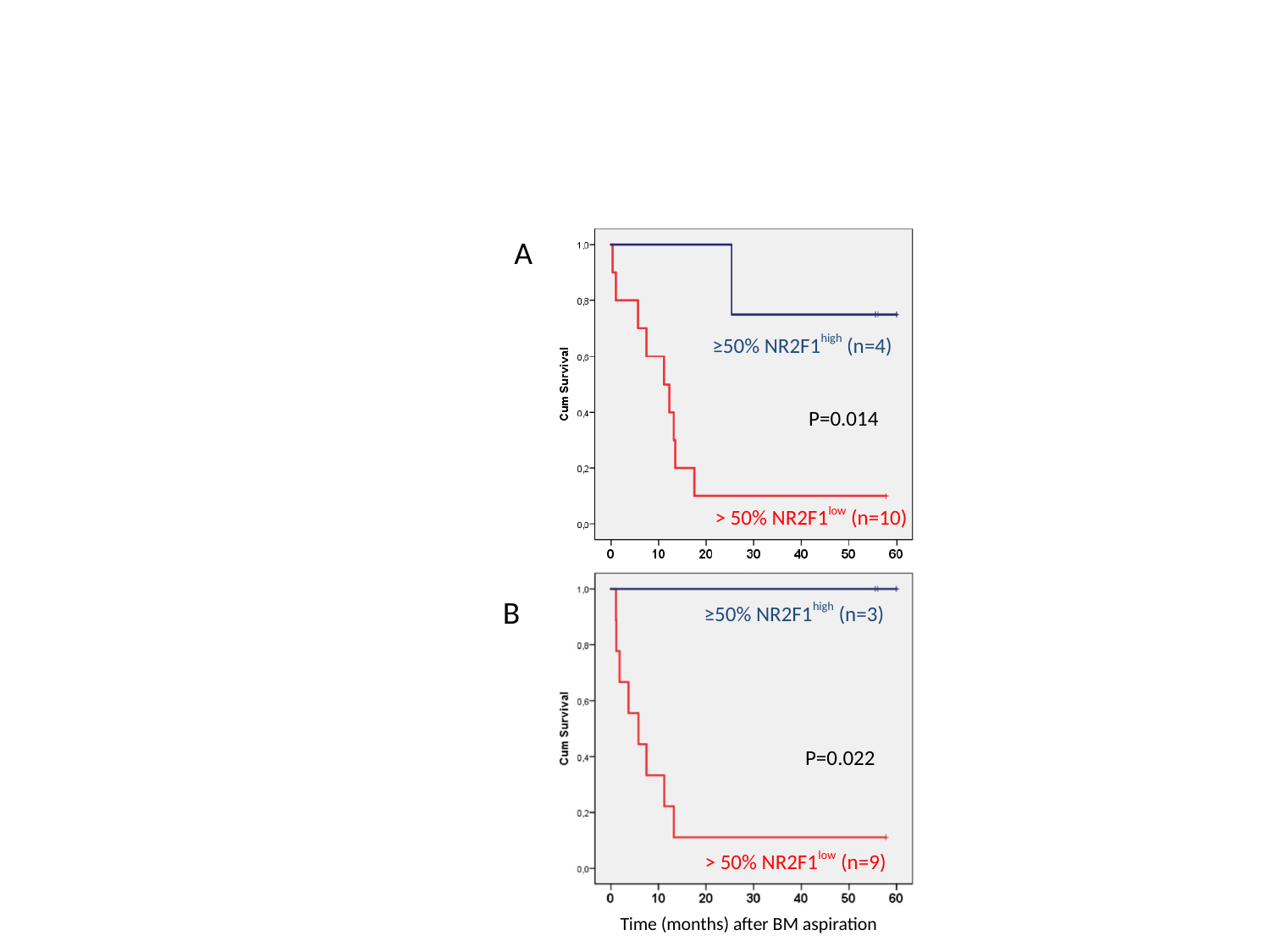

A
≥50% NR2F1high (n=4)
P=0.014
> 50% NR2F1low (n=10)
B
≥50% NR2F1high (n=3)
P=0.022
> 50% NR2F1low (n=9)
Time (months) after BM aspiration

Supplement: Supplementary file 9 — Figure S4. (A) Survival analyses (time to systemic relapse/breast cancer death) in relation to NR2F1 profile of DTCs for patients being nonmetastatic at the time point of last DIF DTC-positive BMA and having no subsequent BM analyzed; patients harboring ≥ 500 DTC excluded. (B) Survival analyses (time to systemic relapse/breast cancer death) in relation to NR2F1 profile of DTCs for patients being nonmetastatic at time point of last DIF DTC-positive BMA and having no subsequent BM analyzed; NeoTax study patients excluded. (PPTX 114 kb) [file 13058_2018_1049_MOESM9_ESM.pptx]

## Slide 1
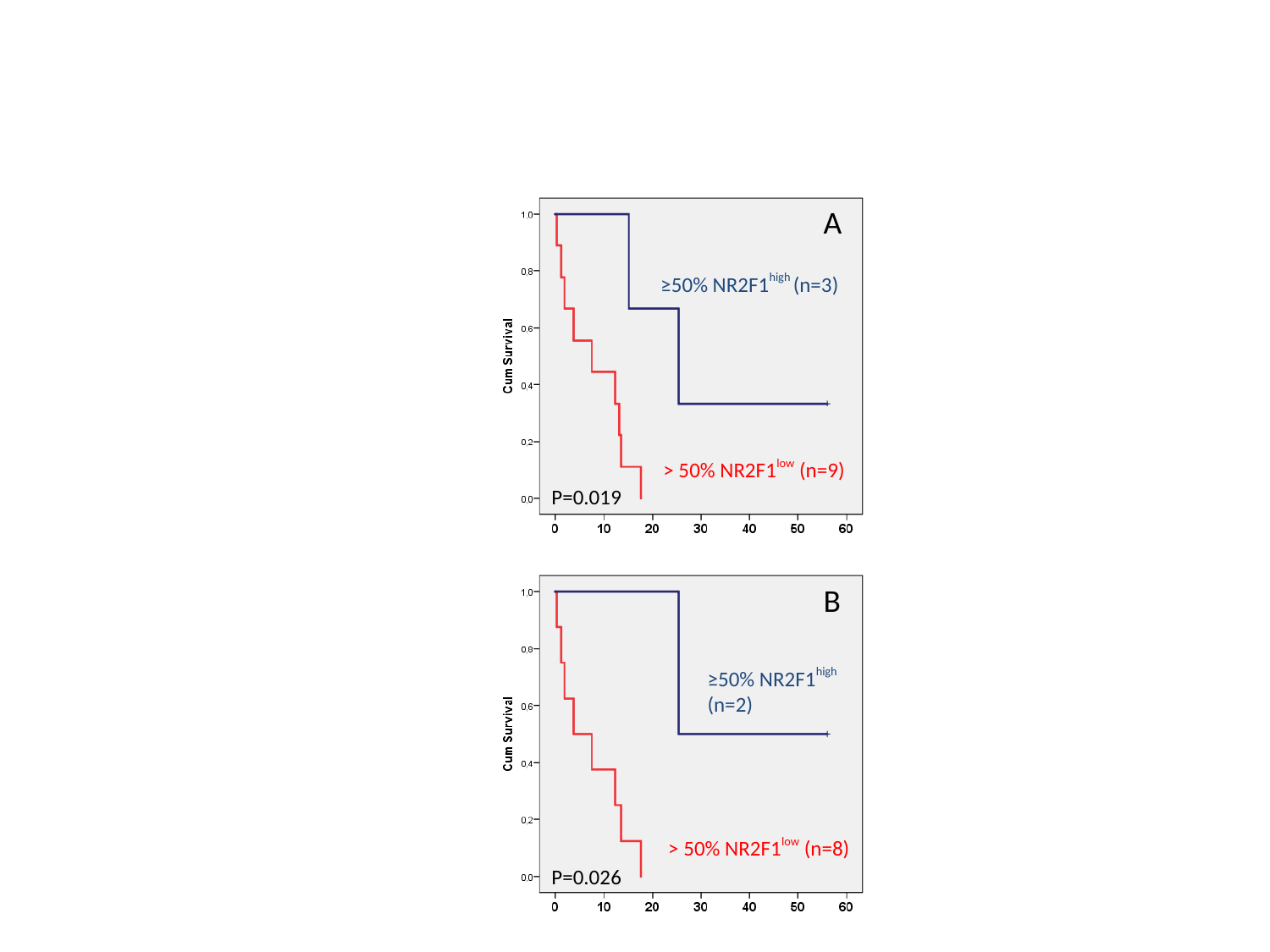

A
≥50% NR2F1high (n=3)
> 50% NR2F1low (n=9)
P=0.019
B
≥50% NR2F1high
(n=2)
> 50% NR2F1low (n=8)
P=0.026

Supplement: Supplementary file 11 — Figure S6. (A) Survival analyses (time to systemic relapse/breast cancer death) in relation to NR2F1 profile at last DIF DTC-positive BMA, restricted to those with Ki67 DTC analysis available (only patients being nonmetastatic at last DIF DTC-positive BMA included). (B) As A, but analysis restricted to patients having no subsequent BMA analyzed. (PPTX 68 kb) [file 13058_2018_1049_MOESM11_ESM.pptx]
