# Supplementary material for: NR2F1 stratifies dormant disseminated tumor cells in breast cancer patients
Source: Breast Cancer Res. 2018 Oct 16;20:120. doi: 10.1186/s13058-018-1049-0 (PMC6190561; doi:10.1186/s13058-018-1049-0)
Supplement: Supplementary file 4 — Table S2. Overview of patient material and DTC results. (DOCX 34 kb) [file 13058_2018_1049_MOESM4_ESM.docx]

**Additional File 2 Table S2.**

**Overview of patient material and DTC results**

| Patients | Number of patients analysed | Samples | Number of samples analysed | Number of CK-positive samples by double IF analysis (%) |
| --- | --- | --- | --- | --- |
| Pts with originally DTC positive status in at least one BM sample | 86 | Cytospins from originally DTC-pos BM-samples | 103 | 32 (31%) |
|  |  | Cytospins from originally DTC-neg BM samples | 11 | 1* (4.2%) |
| Pts with originally DTC negative status in all samples | 11 |  | 13 |  |

***One DTC positive cell detected in one patient with originally DTC negative status**
