# Supplementary material for: NR2F1 stratifies dormant disseminated tumor cells in breast cancer patients
Source: Breast Cancer Res. 2018 Oct 16;20:120. doi: 10.1186/s13058-018-1049-0 (PMC6190561; doi:10.1186/s13058-018-1049-0)
Supplement: Supplementary file 5 — Table S3. Characteristics of the DTC-positive cases by double immunofluorescence (DIF). (DOCX 33 kb) [file 13058_2018_1049_MOESM5_ESM.docx]

**Additional File 3 Table S3.**

**Characteristics of the DTC positive cases by double immunofluorescence (DIF)**

| DIF analysis of patients | Number of pts | % of all originally DTC-pos pts | % of DIF DTC-pos pts |
| --- | --- | --- | --- |
| Detectable DTCs | 26 | 30% |  |
| ≥3 detectable DTCs | 24 | 28% | 92% |
| ≥10 detectable DTCs | 17* | 20% | 67% |
| DTC status analyzed at ≥2 time points | 24 | 29% | 92% |

*Result in the first of two positive BM analysis
