# Supplementary material for: NR2F1 stratifies dormant disseminated tumor cells in breast cancer patients
Source: Breast Cancer Res. 2018 Oct 16;20:120. doi: 10.1186/s13058-018-1049-0 (PMC6190561; doi:10.1186/s13058-018-1049-0)
Supplement: Supplementary file 7 — Table S4. Overview of received treatment between the two BM aspiration time points for the patients presented in Fig. 3. (DOCX 33 kb) [file 13058_2018_1049_MOESM7_ESM.docx]

**Additional File 4 Table S4.**

**Overview of received treatment between the two BM aspiration time points for the patients presented in Figure 3.**

| Patient ID | Treatment between the BM aspiration time points | Comment |
| --- | --- | --- |
| 4 | Chemotherapy +/- endocrine treatment |  |
| 5 | Endocrine treatment only |  |
| 6 | No treatment |  |
| 11 | No treatment |  |
| 20 | Chemotherapy +/- endocrine treatment |  |
| 27 | Chemotherapy +/- endocrine treatment |  |
| 34 | Chemotherapy +/- endocrine treatment |  |
| 35 | Chemotherapy +/- endocrine treatment |  |
| 36 | Chemotherapy +/- endocrine treatment |  |
| 48 | Chemotherapy +/- endocrine treatment |  |
| 57 | Chemotherapy +/- endocrine treatment |  |
| 58 | Chemotherapy +/- endocrine treatment | No DTCs detected |
| 60 | Chemotherapy +/- endocrine treatment |  |
| 61 | Chemotherapy +/- endocrine treatment | No DTCs detected |
| 64 | Endocrine treatment only | No DTCs detected |
| 66 | Chemotherapy +/- endocrine treatment |  |
| 67 | Endocrine treatment only | No DTCs detected |
| 69 | No treatment |  |
| 71 | Chemotherapy +/- endocrine treatment | No DTCs detected |
| 74 | Chemotherapy +/- endocrine treatment |  |
| 78 | Endocrine treatment only |  |
| 82 | Chemotherapy +/- endocrine treatment | No DTCs detected |
| 84 | Chemotherapy +/- endocrine treatment |  |
| 85 | Endocrine treatment only |  |
