# Supplementary material for: NR2F1 stratifies dormant disseminated tumor cells in breast cancer patients
Source: Breast Cancer Res. 2018 Oct 16;20:120. doi: 10.1186/s13058-018-1049-0 (PMC6190561; doi:10.1186/s13058-018-1049-0)
Supplement: Supplementary file 8 — Table S5. Additional results from the serial DTC analyses on samples presented in Fig. 3b and c (in the same order). (DOCX 39 kb) [file 13058_2018_1049_MOESM8_ESM.docx]

**Additional File 5 Table S5. Additional results from the serial DTC analyses on samples presented in Figure 3B and 3C (in the same order)**

|  | Patient ID | **Time after last BM (months) to BrCa event or last FU** | **Metastasis/BrCa death** | **Metastasis before last BM** | **Chemo after last BM** | **% NR2F1^high^ DTCs**  **(last BM)** | **% Ki67 pos DTCs**  **(last BM)** | **# of DIF pos DTCs  (last BM)** | **# of DIF pos DTCs**  **(first BM)** | **# of DTCs by original analysis**  **(last BM)** | **# of DTCs by original analysis (first BM)** |
| --- | --- | --- | --- | --- | --- | --- | --- | --- | --- | --- | --- |
| Fig 3 A | 69 | 15.10 | Yes |  | Yes | 65.4 | 0 | 52 | 8 | 46 | 12 |
|  | 36 | 25.33 | Yes |  |  | 50.0 | 50.0 | 6 | 5 | 8 | 3 |
|  | 35 | NA | Yes | Yes |  | 14.3 | 25.0 | 7 | 19 | 8 | 221 |
|  | 60 | 1.84 | Yes |  |  | 0 | 26.2 | 1000 | 54 | 1000 | 476 |
|  | 27 | 5.69 | Yes |  |  | 0 | ND | 5 | 6 | 10 | 5 |
|  | 4 | 7.50 | Yes |  |  | 0 | 21.7 | 9 | 3 | 86 | 15 |
| Fig 3 B | 57 | 59.93 | No |  |  | 100 | ND | 6 | 0 | 1 | 1 |
|  | 6 | NA | Yes | Yes |  | 94.9 | 1.0 | 233 | 0 | 702 | 0 |
|  | 85 | 56.02 | No |  | Yes | 56.6 | 10.0 | 106 | 0 | 68 | 0 |
|  | 84 | 55.53 | No |  | Yes | 50.0 | ND | 2 | 0 | 2 | 2 |
|  | 48 | 12.24 | Yes |  |  | 25.5 | 20.0 | 17 | 0 | 22 | 0 |
|  | 11 | 3.72 | Yes |  |  | 1.0 | 66.7 | 500 | 0 | 2600 | 0 |
|  | 5 | 1.15 | Yes |  |  | 0 | 15.0 | 1000 | 0 | >1000 | 3 |
|  | 20 | 0.30 | Yes |  |  | 0 | 28.6 | 35 | 0 | 29 | 1 |
|  | 74 | 1.09 | Yes |  |  | 0 | ND | 3 | 0 | 1 | 2 |
|  | 78 | 13.19 | Yes |  | Yes | 0 | 13.8 | 26 | 0 | 55 | 1 |
