# Supplementary material for: NR2F1 stratifies dormant disseminated tumor cells in breast cancer patients
Source: Breast Cancer Res. 2018 Oct 16;20:120. doi: 10.1186/s13058-018-1049-0 (PMC6190561; doi:10.1186/s13058-018-1049-0)
Supplement: Supplementary file 10 — Figure S5. Survival analyses according to NR2F1 and Ki67 DTC profiles of patients being nonmetastatic at the time of last DIF DTC-positive BMA, having no subsequent BM analyzed, and no chemotherapy after last BMA. (PPTX 120 kb) [file 13058_2018_1049_MOESM10_ESM.pptx]

## Slide 1
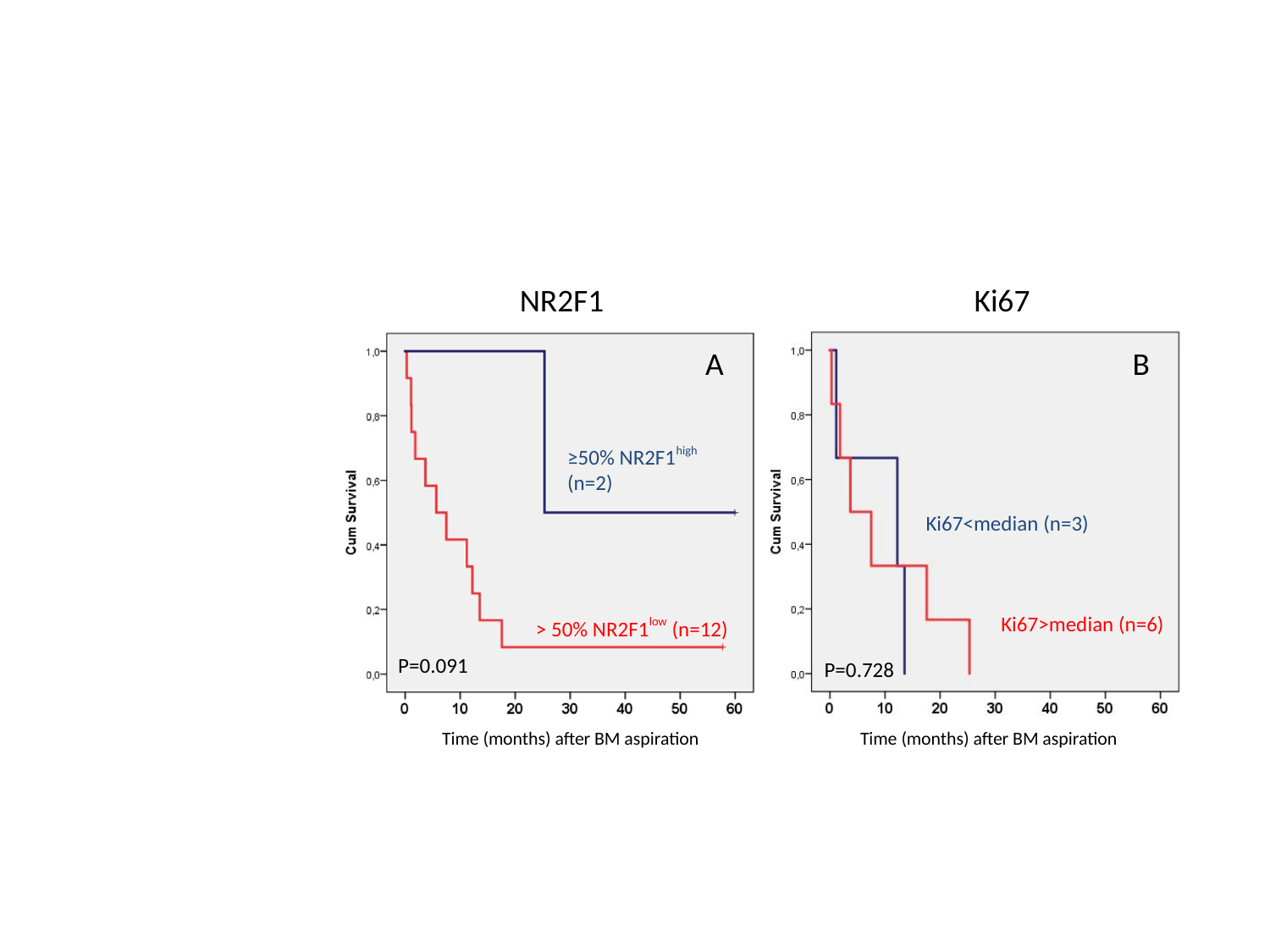

NR2F1
Ki67
B
A
≥50% NR2F1high
(n=2)
Ki67<median (n=3)
Ki67>median (n=6)
> 50% NR2F1low (n=12)
P=0.091
P=0.728
Time (months) after BM aspiration
Time (months) after BM aspiration
